# Acute appendicitis in the COVID-19 era: A complicated situation?

**DOI:** 10.1016/j.amsu.2021.102536

**Published:** 2021-07-02

**Authors:** Joel M. Bowen, Jonathon R.C. Sheen, Helen Whitmore, Chloe Wright, Kirk Bowling

**Affiliations:** a Specialty Trainee Department of Trauma & Orthopaedics, Torbay and South Devon NHS Foundation Trust, UK; b Specialty Trainee Department of Colorectal Surgery, Torbay and South Devon NHS Foundation Trust, UK; c Consultant Department of Upper G.I. Surgery, Torbay and South Devon NHS Foundation Trust, UK; d Trust Doctor Department of General Surgery, Torbay and South Devon NHS Foundation Trust, UK

## Abstract

• Acute appendicitis is the most common intra-abdominal surgical emergency in the world.
• Null hypothesis of no difference in the number of cases of complicated appendicitis.
• What impact did the government's ‘Stay at Home’ message have?
• Increasing body of evidence to support non-operative treatment in certain cases.
• A significantly higher rate of complicated cases found in the COVID era.

## 1 Introduction

The rise and rapid spread of the COVID-19 virus, and subsequent pandemic can be seen to have impacted the health of millions, both directly through infection and indirectly, via numerous factors that have led to delayed treatment of non-COVID related pathology. As a specific example, Tankel et al. showed a 40.7% decrease in the weekly incidence of acute appendicitis, presenting across several centres in Israel, in the first weeks of onset of the COVID-19 pandemic [1]. While the attention of the world and much of the healthcare sector was focussed on the novel virus, other more familiar pathologies did not wait for us to catch up.

Acute appendicitis is a common surgical pathology and the most common intra-abdominal emergency in the world [2]. Despite this, the underlying causative pathology is not always clear, and is frequently misunderstood [3]. Within Western Europe, the prevalence has been more or less stable since the year 2000, at 151 per 100,000 person years [4]. The presentation of acute appendicitis is varied, ranging from subclinical and self-resolving to overwhelming sepsis and death. It is estimated that perforated appendicitis is associated with a 5% mortality rate. This is a figure that is vastly different to the mortality rate associated with “acute but not gangrenous” appendicitis, which is 0.1% [5,6]. Due to this spectrum of severity, it is common practice to divide appendicitis into ‘uncomplicated’ and ‘complicated’ categories. Definitions and methods of grouping vary and are the subject of many years of debate [7]. This is beyond the scope of our paper, and our accepted definitions are ‘uncomplicated’ to include simple, focal or suppurative, and ‘complicated’ to include a gangrenous or perforated appendix and any displaying peritonitis or periappendiceal abscess formation [8].

It is well established that an increased time from symptom onset to operative management increases both the risk of progression of pathology and of complications in the post-operative period [8,9]. Existing literature strongly suggests that delaying appendicectomy is associated with a higher rate of complicated appendicitis, and that this is the progression responsible for the subsequently greater morbidity and mortality reported[[8], [9], [10]].

This study aims to investigate the impact of the COVID-19 pandemic on the nature of pathology found in patients undergoing appendicectomy for acute appendicitis at our centre.

## 2 Methods

### 2.1 Study design

We performed a retrospective study of appendicectomies performed at our centre between March 11, 2019 to March 10, 2021.

Patient data was obtained from our theatre database, followed by patient notes review. Our initial database included all patients who had been treated with operative appendicectomy, both laparoscopic and open. For each patient, we reviewed their admission document, discharge summary, operation note, radiology reports and histology findings.

Patients were then grouped into Group 1 (‘pre-COVID’) - March 11, 2019 to March 10, 2020 - and Group 2 (‘COVID’) - March 11, 2020 to March 10, 2021. We used the March 11, 2020 as our distinguishing date between the two groups, as this was the date on which the World Health Organisation declared the COVID-19 pandemic [11].

### 2.2 Exclusion criteria

Patients were excluded from analysis if the appendicectomy was performed as an elective procedure (either as an interval appendicectomy or as part of another elective operation, eg hysterectomy or oophorectomy), if the appendix was removed during an emergency procedure not due to appendicitis or if the histology sample was missing.

### 2.3 Statistical analysis

Anonymised data was gathered into a single spreadsheet using Microsoft Excel 2019. The null hypothesis of no difference in the number of cases of complicated appendicitis during the first year of COVID-19 vs the year prior, was analysed using a two-tailed Fisher's exact test, to a p value < 0.05.

### 2.4 Ethical considerations

Given the nature of this study, ethical approval was neither required nor sort. Our work has been reported based on The STROCSS 2019 Guideline: Strengthening the Reporting of Cohort Studies in Surgery [12]. This study is registered with the ResearchRegistry and the unique identifying number is: researchregistry6927 [13].

## 3 Results

**Fig. 1:**
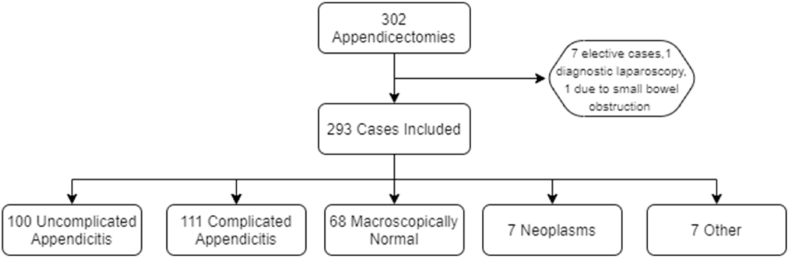
Flow diagram for Group 1 cases (March 11, 2019–March 10, 2020).

**Fig. 2:**
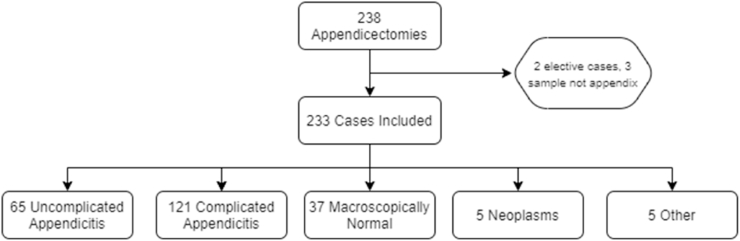
Flow diagram for Group 2 cases (March 11, 2020–March 10, 2021).

From March 11, 2019 to March 10, 2021, 540 appendicectomies were performed at our centre - 302 cases in Group 1 and 238 cases in Group 2. In Group 1, 9 cases were then excluded - 7 due to being elective procedures, 1 case as the appendix was removed due to adhesions causing small bowel obstruction, and 1 case in which only a diagnostic laparoscopy was performed - bringing the total cases to 293 (See Fig. 1). In Group 2, 5 cases were excluded - 2 due to being elective procedures and 3 due to there being no appendiceal histological sample - bringing the total number of cases to 233 (See Fig. 2).

Within Group 1, the age range was 6–85 years, with a mean of 35.59 years, whilst in Group 2, the age range was 6–87 years, with a mean of 33.95 years.

After full review of patient notes, and referring to the definitions of uncomplicated and complicated appendicitis outlined in the Introduction, Group 1 consisted of 100 cases of uncomplicated appendicitis, 111 of complicated appendicitis, 68 macroscopically normal, 7 neoplastic and 7 others. Group 2 contained 65 cases of uncomplicated appendicitis, 121 complicated, 37 macroscopically normal, 5 neoplastic and 5 others.

Given the differing population sizes of the two groups, it was pertinent to compare the proportional variation across the groups. In Group 1, complicated appendicitis accounted for 37.9% of cases (111/293) and uncomplicated appendicitis accounted for 34.1% of cases (100/293). In Group 2, complicated appendicitis accounted for 51.9% of cases (121/233) and uncomplicated appendicitis accounted for 27.9% of cases (65/233).

Fisher's exact test was utilised to assess our null hypothesis. This was tested by first making the comparison between complicated appendicitis and all other emergency appendicectomies, and then by comparison of complicated appendicitis with uncomplicated appendicitis (See Table 1, Table 2). Within each of these comparisons, the Fisher's exact test statistical value was <0.00001, meaning the result was significant at p < 0.05, thus disproving our null hypothesis.

**Table 1:** Fisher's exact test data for complicated appendicitis vs all other cases.

	COMPLICATED APPENDICITIS	ALL OTHER CASES	TOTAL
PRE-COVID	37.9%	62.1%	100%
COVID	51.9%	48.1%	100%
TOTAL	89.8%	110.2%

**Table 2:** Fisher's exact test data for complicated appendicitis vs uncomplicated appendicitis.

	COMPLICATED APPENDICITIS	UNCOMPLICATED APPENDICITIS	TOTAL
PRE-COVID	37.9%	34.1%	72.0%
COVID	51.9%	27.9%	79.8%
TOTAL	89.8%	62.0%	

## 4 Discussion

Our results demonstrate a significantly higher incidence of complicated appendicitis in patients who underwent appendicectomy during the COVID-19 era, when compared to those in the prior year. The groups were reasonably homogenous, with differing group size accounted for, similar mean age, and exclusion rates low in both. To the best of our knowledge, this is the first study to compare an entire year of data from the pre-COVID to the COVID era in relation to cases of acute appendicitis. Previous studies carried out across a shorter timeframe have however reported comparable findings to our own [1,[14], [15], [16], [17], [18]]. We propose a few possible explanations for the significant difference found across the two groups contained within our study.

Firstly, guidance outlined by Public Health England and government policy in the UK heavily emphasised a “stay at home” message, strongly advising against any unnecessary travel. In the early days of the pandemic, daily briefings from Downing Street reinforced the gravity of the situation, and media reporting of infection and death rates spread a culture of fear across the nation. It is possible that these factors may have caused a proportion of patients, due to a desire to follow advice from the authorities, to present later in the evolution of an acute appendicitis than they would have in the pre-COVID era.

A strong message to stay at home and “protect the NHS” was well publicised during this time period. Compounded by concerns about hospitals being overrun, and widely shared images of intensive care units full of patients on ventilators, it seems plausible that a belief spread that help would not be accessible for any other health condition. Equally, patients developing symptoms of early appendicitis may have feared contracting the virus if they presented to hospital, or felt that resources were better allocated to others ‘more in need’. Any combination of these factors could have contributed to delayed presentation, and the higher proportion of complicated appendicitis that we have identified.

In early 2020, advice was issued by the Royal College of Surgeons to consider non-operative treatment of appendicitis where “possible and reasonable (such as for early appendicitis)”, and to carefully select for laparoscopic surgery only those patients in whom benefit “substantially exceeds the risk of potential viral transmission” [19]. This was supported by a management algorithm published by the Association of Upper Gastrointestinal Surgery (AUGIS) of Great Britain and Ireland. Developed early on in the pandemic, this algorithm advocated the non-operative management of all cases of appendicitis, except for those involving perforations [20].

There is a reasonable body of evidence to support non-operative treatment of appendicitis predating the pandemic [21] - most notably several randomised controlled trials, albeit with some result discrepancy, and subsequent meta-analyses of their findings [[22], [23], [24], [25], [26], [27], [28], [29]]. This body of evidence has been reinforced by papers from the early stages of the pandemic [14,15], to provide a recommendation that in the right carefully selected patient, non-operative treatment may provide a reasonable alternative option [[30], [31], [32]]. As such, the unprecedented additional strains and risks of the oncoming pandemic made the selection of non-operative treatment of appendicitis a legitimate strategy.

It must be taken into consideration that our study has looked only at those patients who underwent operative management of their condition. This is a potential source of bias in our results, as it could be proposed that those cases treated non-operatively, were more likely to be uncomplicated [33]. To address this limitation would require a study investigating all diagnosed cases during a given period, but positive identification of these subjects could prove problematic in a healthcare system such as the UK's NHS, where a significant proportion of cases are diagnosed clinically and confirmed only on laparoscopy or subsequent histology.

A further limitation of our study is one inherent in our design, as a single centre cohort. Whilst pragmatic, this approach limits the extent to which our findings can be generalised to elsewhere within the UK or internationally. The geographical location of our 300-bed hospital is one that was relatively spared by COVID-19 compared to the UK average, with 4260 total cases (as of 15/05/21) and 153 COVID-19 related deaths (as of 15/05/21) [34]. It is difficult to quantify the impact that this fact may have on the generalisability of our findings, as this knowledge could have influenced the behaviour of our local population to have been bolder in their access to secondary care, but could equally have had a negligible impact in the age of global media and sharing of information; or indeed, misinformation. It should also be noted that our centre serves an ageing population, with an average age over 5 years above national average and a disproportionate representation of over 50's.

## 5 Conclusion

This study was conducted with the aim of comparing the proportion of complicated to uncomplicated appendicitis since the onset of the COVID-19 pandemic to the previous year. As outlined, we have discovered a significantly higher rate of complicated cases in the COVID era. The question that resonates given these findings is whether this situation was in any way avoidable. As discussed, this common condition retains a significant rate of morbidity and mortality, particularly in the frail and comorbid. Given that many of these patients would have been acutely aware of their vulnerability to COVID-19, we question whether enough was done to maintain accessibility of our services throughout a strange and at times unnerving first year of the pandemic. Or was access always there, but the overwhelming fear generated by the novel virus, and the attention that it received, the true culprit and barrier to prompt surgical care?

COVID-19 continues to impact lives across the globe daily. As it does, we must continue to evolve in our ability to work around it, and this is clearly of paramount importance in the healthcare sector. We recommend further study on a national or international scale to test our findings, and from the growing body of literature, we hope to contribute to decision making and strategic planning. In combination with global studies such as the WHO's independent investigation into COVID-19 management, we can make informed decisions going forward. Our institution is performing ongoing risk analysis and has active projects in place publicising the safety of access to our health service, to address concerns such as those raised here.

As this situation continues to develop, one of the major challenges that we must now face is that of applying the lessons learnt in one aspect of our practice to another. The issues and principles outlined by our findings have applicability far beyond just appendicitis, as the impact of this pandemic has been felt in some way across the vast spectrum of medical and surgical pathologies.

## Provenance and peer review

Not commissioned, externally peer-reviewed.

## Compliance with ethical standards

This work has been reported based on The STROCSS 2019 Guideline: Strengthening the Reporting of Cohort Studies in Surgery.

## Sources of funding

The authors declare that sponsors had no involvement in this study.

## Ethical approval

Given the nature of this study, ethical approval was not required, nor sought.

## Consent

Given the retrospective nature of the study and anonymised data presentation, informed consent was not required or obtained.

## Registration of research studies

This study is registered with the ResearchRegistry and the unique identifying number is: researchregistry6927.

https://www.researchregistry.com/browse-the-registry#home/registrationdetails/60d8f1c45dbeef001e8bee5f/

## Author contribution

JMB: study conception, data collection, interpretation and analysis, manuscript preparation.

JRCS: study conception, data collection, interpretation and analysis, manuscript preparation.

HW: data collection, interpretation and analysis, manuscript preparation.

CW: data collection.

KBB: study conception, critical revision of manuscript.

## Guarantor

Jonathon Sheen.

## Declaration of competing interest

The authors declare that there is no conflict of interests regarding the publication of this paper.

## References

[1] Tankel J., Keinan A., Blich O., Koussa M., Helou B., Shay S., Zugayar D., Pikarsky A., Mazeh H., Spira R., Reissman P. (2020 Aug). The decreasing incidence of acute appendicitis during COVID-19: a retrospective multi-centre study. World J. Surg..

[2] Baird D.L.H., Simillis C., Konto Vouniseas C. (2017). Acute appendicitis. BMJ.

[3] Carr Norman J. (2000). The pathology of acute appendicitis. Ann. Diagn. Pathol..

[4] Ferris M., Quan S., Kaplan B.S. (2017). The global incidence of appendicitis: a systematic review of population-based studies. Ann. Surg..

[5] Flum D.R. (2015 May 14). Clinical practice. Acute appendicitis--appendectomy or the "antibiotics first" strategy. N. Engl. J. Med..

[6] Di Saverio S. (2020 Apr 15). Diagnosis and treatment of acute appendicitis: 2020 update of the WSES Jerusalem guidelines. World J. Emerg. Surg..

[7] Buonpane C., Goldstein S., Hunter C. (2019). Defining the disease: uncomplicated versus complicated appendicitis. Controversies in Pediatric Appendicitis.

[8] Kim M., Kim S.J., Cho H.J. (2016). Effect of surgical timing and outcomes for appendicitis severity. Ann. Surg. Treat. Res..

[9] Ditillo M.F., Dziura J.D., Rabinovici R. (2006). Is it safe to delay appendectomy in adults with acute appendicitis?. Ann. Surg..

[10] Jeon B.G., Kim H.J., Jung K.H., Lim H.I., Kim S.W., Park J.S., Kim K.H., Kim I.D. (2016 Jan). Appendectomy: should it Be performed so quickly?. Am. Surg..

[11] WHO Director-General's Opening Remarks at the Media Briefing on COVID-19 - 11 March 2020.

[12] Agha R., Abdall-Razak A., Crossley E., Dowlut N., Iosifidis C., Mathew G., for the STROCSS Group (2019). The STROCSS 2019 guideline: strengthening the reporting of cohort studies in surgery. Int. J. Surg..

[13] https://www.researchregistry.com/browse-the-registry#home/registrationdetails/60d8f1c45dbeef001e8bee5f/.

[14] Neufeld M.Y., Bauerle W., Eriksson E. (2021). Where did the patients go? Changes in acute appendicitis presentation and severity of illness during the coronavirus disease 2019 pandemic: a retrospective cohort study. Surgery.

[15] Javanmard-Emamghissi H., Boyd-Carson H., Hollyman M. (2021). The management of adult appendicitis during the COVID-19 pandemic: an interim analysis of a UK cohort study. Tech. Coloproctol..

[16] Basamh M., Rajendiran A., Chung W.Y., Runau F., Sangal S. (2020). Management of appendicitis during the COVID pandemic: lessons from the first month of the outbreak. Br. J. Surg..

[17] Orthopoulos G., Santone E., Izzo F. (2020). Increasing incidence of complicated appendicitis during COVID-19 pandemic [published online ahead of print, 2020 Sep 28]. Am. J. Surg..

[18] Finkelstein Paige, Omar Picado, Muddasani Kiranmayi, Henry Wodnicki, Mesko Thomas, Unger Stephen, Bao Philip, Jorge Irving, Narayanan Sumana, Ben-David Kfir (2021). A retrospective analysis of the trends in acute appendicitis during the COVID-19 pandemic. J. Laparoendosc. Adv. Surg. Tech..

[19] Association of Surgeons of Great Britain & Ireland (2020). Association of coloproctology of Great Britain & Ireland, association of upper gastrointestinal Surgeons, royal College of Surgeons of edinburgh, royal College of Surgeons of England, royal College of physicians and Surgeons of glasgow, royal College of Surgeons in Ireland. Updated Intercollegiate General Surgery Guidance on COVID‐19.

[20] https://www.augis.org/wp-content/uploads/2020/04/AA-Management-Pathway-During-Covid-19.pdf.

[21] Findlay J.M. (2020). Managing appendicitis during the COVID‐19 pandemic—what do we need to know from the evidence?. Int. J. Clin. Pract..

[22] Eriksson S., Granstrom L. (1995). Randomized controlled trial of appendicectomy versus antibiotic therapy for acute appendicitis. Br. J. Surg..

[23] Styrud J., Eriksson S., Nilsson I. (2006). Appendectomy versus antibiotic treatment in acute appendicitis. A prospective multicenter randomized controlled trial. World J. Surg..

[24] Hansson J., Korner U., Khorram‐Manesh A., Solberg A., Lundholm K. (2009). Randomized clinical trial of antibiotic therapy versus appendicectomy as primary treatment of acute appendicitis in unselected patients. Br. J. Surg..

[25] Turhan A.N., Kapan S., Kutukcu E., Yigitbas H., Hatipoglu S., Aygun E. (2009). Comparison of operative and non operative management of acute appendicitis. Ulus. Travma Acil. Cerrahi Derg. [Turk. J. Trauma Emerg. Surg.].

[26] Vons C., Barry C., Maitre S. (2011). Amoxicillin plus clavulanic acid versus appendicectomy for treatment of acute uncomplicated appendicitis: an open‐label, non‐inferiority, randomised controlled trial. Lancet.

[27] Salminen P., Paajanen H., Rautio T. (2015). Antibiotic therapy vs appendectomy for treatment of uncomplicated acute appendicitis: the APPAC randomized clinical trial. J. Am. Med. Assoc..

[28] Park H.C., Kim M.J., Lee B.H. (2016). Randomized clinical trial of antibiotic therapy for uncomplicated appendicitis. Br. J. Surg..

[29] Talan D.A., Saltzman D.J., Mower W.R. (2017). Olive View–UCLA Appendicitis Study Group. Antibiotics‐first versus surgery for appendicitis: a US pilot randomized controlled trial allowing outpatient antibiotic management. Ann. Emerg. Med..

[30] Mason R.J., Moazzez A., Sohn H., Katkhouda N. (2012). Meta‐analysis of randomized trials comparing antibiotic therapy with appendectomy for acute uncomplicated (no abscess or phlegmon) appendicitis. Surg. Infect..

[31] Varadhan K.K., Neal K.R., Lobo D.N. (2012). Safety and efficacy of antibiotics compared with appendicectomy for treatment of uncomplicated acute appendicitis: meta‐analysis of randomised controlled trials. BMJ.

[32] Findlay J.M., el Kafsi J., Hammer C., Gilmour J., Gillies R.S., Maynard N.D. (2016). Nonoperative management of appendicitis in adults: a systematic review and meta‐analysis of randomized controlled trials. J. Am. Coll. Surg..

[33] Podda M., Pata F., Pellino G., Ielpo B., Di Saverio S. (2021). Acute appendicitis during the COVID-19 lockdown: never waste a crisis!. Br. J. Surg..

[34] https://coronavirus.data.gov.uk.

